# Quantitative meta-analysis of neural activity in posttraumatic stress disorder

**DOI:** 10.1186/2045-5380-2-9

**Published:** 2012-05-18

**Authors:** Jasmeet P Hayes, Scott M Hayes, Amanda M Mikedis

**Affiliations:** 1 National Center for PTSD, VA Boston Healthcare System, Boston, MA, USA; 2 Neuroimaging Research Center, VA Boston Healthcare System, Boston, MA, USA; 3 Department of Psychiatry, Boston University School of Medicine, Boston, MA, USA; 4 Memory Disorders Research Center, VA Boston Healthcare System and Boston University School of Medicine, Boston, MA, USA

**Keywords:** Activation likelihood estimation, fMRI, PET, Amygdala, Anterior cingulate cortex, Ventromedial prefrontal cortex, Salience network, Fear conditioning

## Abstract

**Background:**

In recent years, neuroimaging techniques such as functional magnetic resonance imaging (fMRI) and positron emission tomography (PET) have played a significant role in elucidating the neural underpinnings of posttraumatic stress disorder (PTSD). However, a detailed understanding of the neural regions implicated in the disorder remains incomplete because of considerable variability in findings across studies. The aim of this meta-analysis was to identify consistent patterns of neural activity across neuroimaging study designs in PTSD to improve understanding of the neurocircuitry of PTSD.

**Methods:**

We conducted a literature search for PET and fMRI studies of PTSD that were published before February 2011. The article search resulted in 79 functional neuroimaging PTSD studies. Data from 26 PTSD peer-reviewed neuroimaging articles reporting results from 342 adult patients and 342 adult controls were included. Peak activation coordinates from selected articles were used to generate activation likelihood estimate maps separately for symptom provocation and cognitive-emotional studies of PTSD. A separate meta-analysis examined the coupling between ventromedial prefrontal cortex and amygdala activity in patients.

****Results**:**

Results demonstrated that the regions most consistently hyperactivated in PTSD patients included mid- and dorsal anterior cingulate cortex, and when ROI studies were included, bilateral amygdala. By contrast, widespread hypoactivity was observed in PTSD including the ventromedial prefrontal cortex and the inferior frontal gyrus. Furthermore, decreased ventromedial prefrontal cortex activity was associated with increased amygdala activity.

****Conclusions**:**

These results provide evidence for a neurocircuitry model of PTSD that emphasizes alteration in neural networks important for salience detection and emotion regulation.

## **Background**

In the aftermath of highly distressing and shocking events such as combat, genocide, and rape, a subset of individuals develop posttraumatic stress disorder (PTSD), which is characterized by distressing memories of the event, physiological hyperarousal, and impairment in daily functioning. With the growing interest in PTSD due in part to its high prevalence among veterans of the Iraq and Afghanistan wars, there is an urgency to understand the neural pathogenesis of the disorder. Neuroimaging studies have been conducted to examine brain regions involved in PTSD [1-26]. Based on these findings and the non-human animal literature, the prevailing neurocircuitry model of PTSD suggests that PTSD can be understood in terms of circuits involved in fear conditioning in the brain. Specifically, this model suggests that heightened amygdala activity gives privileged status to feared and threatening stimuli. Whereas the ventromedial prefrontal cortex would normally temper amygdala activity, abnormal function of this region reduces regulation of amygdala output [27]. Furthermore, altered hippocampal function may result in impaired ability to discern safe from dangerous contexts.

The aforementioned brain regions, which play a key role in nonhuman animal fear conditioning [28], likely play an important role in PTSD. PTSD is more likely to develop following highly fear-provoking and life-threatening events than less intense events [29]. Influential psychological theories of PTSD have emphasized the role of fear structures and fear conditioning in the development and maintenance of the disorder [30,31]. Furthermore, exposure therapy, which involves the principles of extinction learning [30], is one of the most effective therapeutic interventions for PTSD.

However, fear conditioning models are limited in their ability to explain the full range of human experience and emotion. Fear conditioning can occur outside of conscious awareness, yet conscious processes such as voluntary and effortful avoidance of thoughts and memories of the trauma play a vital role in the development and maintenance of the disorder [32]. This has led to growing supposition that fear-circuitry models are unable to fully account for the heterogeneity of symptoms following a traumatic event [33] and that anxiety and fear may not be the central components in explaining PTSD symptomatology as previously believed [34]. Accordingly, the proposed revision of the Diagnostic and Statistical Manual (DSM-V) may now recognize negative cognitions and persistent negative mood states as key symptoms of the diagnosis [35], suggesting that other emotions such as dysphoria are important in the development and maintenance of the disorder in addition to fear. Therefore, a primary goal of the present study was to examine patterns of brain activation in neuroimaging studies of PTSD that may provide a more complete understanding of the neural circuitry of PTSD.

In the present study, we performed a quantitative meta-analysis of neuroimaging studies in PTSD using activation likelihood estimation (ALE). This method calculates the probability that a given voxel is activated consistently across studies rather than a single study [36] and therefore provides a more objective measure of brain activity in PTSD than qualitative reviews. Although there have been two prior functional neuroimaging meta-analyses in PTSD [37,38], the present study includes more recent studies, focuses solely on adult PTSD, and considers separately the effects of study type (symptom provocation versus cognitive-emotional) and neuroimaging analysis type (whole-brain voxel-wise analysis versus region-of-interest [ROI] analysis). Symptom provocation studies are designed to elicit trauma-related symptoms whereas cognitive-emotional studies include emotional stimuli (e.g., fearful face) but do not explicitly cue the patient to their traumatic event. In contrast to previous meta-analyses in PTSD, the current study separates symptom provocation and cognitive-emotional studies to examine the neural correlates of two primary characteristics of PTSD: specific recall of a traumatic event (symptom provocation) and emotional response generalization (cognitive-emotional studies). Furthermore, examining results from whole-brain voxel-wise analyses separately from ROI analyses may provide greater insight whether the regions typically targeted in ROI studies (e.g., the amygdala) are also robustly active when taking into account all voxels in the brain. ROI analyses restrict statistical analysis to the small number of a priori defined voxels, reducing the need for more stringent correction for multiple comparisons; thus, ROI studies are not entirely comparable to studies employing whole-brain voxel-wise statistics. In the present study, we examined the results from ROI studies as they comprise a significant proportion of imaging studies in PTSD, with the recognition that whole brain voxel-wise analyses represent a less biased statistical approach. Finally, we performed a separate meta-analysis to test the fear-model hypothesis that hypoactivity in the ventromedial prefrontal cortex is associated with hyperactivity in the amygdala, reflecting insufficient inhibition of prefrontal cortex over the amygdala.

## **Methods**

### **Article selection**

Using keywords “PTSD,” “neuroimaging,” “fMRI,” and “PET,” a literature search in PubMed and Published International Literature on Traumatic Stress (PILOTS) was conducted for PET and fMRI studies of adult PTSD that were published before February 2011. The article search resulted in 79 functional neuroimaging studies. Included studies contrasted a traumatic or negative emotional condition with a resting baseline, positive condition, or neutral condition, conducted between-group analyses using subtraction methodology, and reported between-group peak activation coordinates in standard space. For relevant articles that did not report whole-brain results, the authors were contacted to request activation coordinates [6,10]. Case studies were excluded [39,40] as well as studies examining PTSD and co-morbidity with other disorders, although an exception was made for major depressive disorder (MDD) because of its high co-morbidity with PTSD [13]. Based on these inclusion and exclusion criteria, 26 adult PTSD neuroimaging studies reporting results from 342 patients and 342 controls remained in the analyses (see Table 1).

**Table 1:** Functional neuroimaging studies included in meta-analysis

**Study**	**PTSD**	TC*	NTC**	**Type of trauma**	**Contrast used in meta-analysis**	**Scanning task**	**Imaging method**	**Design**
**Symptom Provocation Whole Brain Analyses (10)**								
Bremner et al. 1999a	10	10		Combat	Combat vs. neutral pictures and sounds	View and listen	PET	Block
Bremner et al. 1999b	10	12		SA^+^	Childhood abuse vs. neutral scripts	Image and remember event	PET	Block
Britton et al. 2005	16	15		Combat	Combat vs. neutral scripts	Listen and maintain evoked emotional state	PET	Block
Hou et al. 2007	10	7		Mining accident	Mining accident vs. neutral pictures	View	fMRI	Block
Lanius et al. 2001	9	9		Mixed	Trauma scripts vs. baseline	Listen and remember event	fMRI	Block
Lanius et al. 2002	7	10		SA (1 MVA) ^+^	Trauma scripts vs. baseline	Listen and remember event	fMRI	Block
Lanius et al. 2003	10	10		Mixed	Trauma scripts vs. baseline	Listen and remember event	fMRI	Block
Lanius et al. 2007	26	16		MVA^+^	Trauma vs. neutral scripts	Listen and remember event	fMRI	Block
Shin et al. 1999	8	8		SA^+^	Sexual abuse vs. neutral scripts	Recall and imagine contents of script	PET	Block
Shin et al. 2004	17	19		Combat	Combat vs. neutral scripts	Recall and imagine contents of script	PET	Block
**Symptom Provocation ROI Analyses (2)**								
Frewen et al. 2008	25	16		MVA^+^	Trauma vs. neutral scripts	Listen to and image script	fMRI	Block
Protopopescu et al. 2005	9	14		SA, PA^+^	PTSD vs. neutral words	Read word	fMRI	Block
**Cognitive-Emotional Whole Brain Analyses (12)**								
Bremner et al. 2003	10		11	SA^+^	Negative emotional vs. neutral word pairs	Declarative memory task	PET	Block
Bremner et al. 2004	12	9		SA^+^	Negative emotional vs. neutral words	Stroop task	PET	Block
Brunetti et al. 2010	10		10	Assault	Negative emotional vs. neutral IAPS pictures	Visuo-attentional task	fMRI	Block
Felmingham et al. 2010	23	21		Mixed	Fearful vs. neutral faces	Backward masking task	fMRI	Block
Fonzo et al. 2010	12		12	IPV^+^	Fearful vs. happy faces	Emotional face matching task	fMRI	Block
Kim et al. 2008	12		12	Fire	Fearful vs. neutral faces	Same-different judgment task	fMRI	Event-related
Sakamoto et al. 2005	16		16	Mixed	Traumatic vs. neutral images	View stimuli below perceptual threshold	fMRI	Block
Shin et al. 2005	13	13		Combat, fire	Fearful vs. happy faces	Overt passive viewing task	fMRI	Block
Thomaes et al. 2009	9		9	SA, PA^+^	Negative words vs. baseline	Word classification task	fMRI	Event-related
Whalley et al. 2009	16	16	16	Mixed	Negative vs. neutral background pictures with neutral foreground pictures	Episodic memory retrieval task	fMRI	Event-related
Williams et al. 2006	13		13	Mixed	Fearful vs. neutral faces	Overt fear perception task	fMRI	Block
Hou et al. 2007	Same Hou et al. 2007 article as the one listed above (in addition to symptom provocation coordinates, article reported coordinates from a short-term memory recall task)							
**Cognitive-Emotional ROI Analyses (6)**								
Bryant et al. 2008	15		15	Mixed	Fearful vs. neutral faces	View stimuli below conscious threshold	fMRI	Block
Phan et al. 2006	16	15		Combat	Negative vs. neutral IAPS pictures	View and rate pictures	PET	Block
Rauch et al. 2000	8	8		Combat	Fearful vs. positive faces	Masked faces paradigm	fMRI	Block
Felmingham et al. 2010	Same as whole brain article above							
Fonzo et al. 2010	Same as whole brain article above							
Williams et al. 2006	Same as whole brain article above							

*TC: trauma exposed controls without PTSD. **NTC: non-traumatized controls.

^*+*^*SA*: sexual abuse/assault; *PA*: physical abuse/assault; *MVA*: motor vehicle accident; *NSA*: non sexual assault; *IPV*: intimate partner violence.

### **Inclusion/exclusion criteria for activation foci**

For each of the articles listed in Table 1, significant peak activation coordinates were extracted for negative > other (baseline, positive, or neutral) between-group contrasts (PTSD > Controls; Controls > PTSD). When coordinates for more than one type of negative > other contrast were reported in the same study, only one contrast was included to avoid using foci from the same participants twice [4,9,16,25]. In these cases, the selected contrast compared a trauma-specific or fear-inducing condition with a neutral condition. If a study conducted a whole-brain and a ROI analysis [8,9,12,26], coordinates from both analyses were included provided that the ROIs were not reported in the whole-brain results [8,9,26].

In studies that included two levels of control groups (e.g., healthy controls and trauma-exposed controls) or PTSD patients (e.g., PTSD with MDD versus PTSD without MDD), only foci from one of the between-group comparisons were used (i.e., between-group foci for PTSD vs. traumatized controls [5,8] and PTSD without co-morbidity vs. controls [13]). Following inclusion and exclusion of coordinates, 218 between-group activation foci remained (Table 2).

**Table 2:** Number of activation foci included in between-group analyses

**Study**	**P > C**^**+**^	**C > P**^**+**^	**Statistical threshold**
**Symptom Provocation Whole Brain Analyses**			
Bremner et al. 1999a	5	5	*P* < .001
Bremner et al. 1999b	9	19	*P* < .001
Britton et al. 2005	--	1	*P* < .005
Hou et al. 2007	1	9	*P* < .005
Lanius et al. 2001	--	4	*P* < .001
Lanius et al. 2002	9	3	*P* < .001, > 10 voxels
Lanius et al. 2003	--	9	*P* < .001, > 10 voxels
Lanius et al. 2007	--	2	*P* < .05 cor., > 10 voxels
Shin et al. 1999	4	14	*P* < .001
Shin et al. 2004	1	3	*P* < .001
**Symptom Provocation ROI Analyses**			
Protopopescu et al. 2005	1	--	*P* < .01 cor.
Frewen et al. 2008	--	3	*P* < .05, > 10 voxels
**Cognitive-Emotional Whole Brain Analyses**			
Bremner et al. 2003	15	14	*P* < .01 cor.
Bremner et al. 2004	2	6	*P* < .005 cor., > 65 voxels
Brunetti et al. 2010	6	3	*P* < .001
Felmingham et al. 2010	1	--	*P* < .001, > 10 voxels
Fonzo et al. 2010	3	1	*P* < .05
Kim et al. 2007	7	6	*P* < .001
Sakamoto et al. 2005	1	4	*P* < .01
Shin et al. 2005	4	4	*P* < .001
Thomaes et al. 2009	2	--	*P* < .001
Whalley et al. 2009	3	1	*P* < .001
Williams et al. 2006	7	1	*P* < .001
Hou et al. 2007*	--	2	*P* < .005
**Cognitive-Emotional ROI Analyses**			
Bryant et al. 2008	4	--	*P* < .05, > 3 voxels
Phan et al. 2006	--	1	*P* < .005 cor.
Rauch et al. 2000	1	--	*P* < .05
Felmingham et al. 2010**	9	--	*P* < .001, > 10 voxels
Fonzo et al. 2010**	2	--	*P* < .05
Williams et al. 2006**	2	4	*P* < .001
**Total SP foci**	**30**	**72**	
**Total cognitive-emotional foci**	**69**	**47**	
**Total number of foci**	**99**	**119**	

^+^*P > C*: PTSD patients > Controls; *C > P*: Controls > PTSD patients.

*Paper also reported symptom provocation coordinates.

**Papers also reported whole brain coordinates.

*cor.* = corrected for multiple comparisons.

### **Meta-analyses**

Coordinate-based random-effects meta-analyses were conducted using GingerALE software version 2.1 (http://brainmap.org/ale/). Coordinates reported in MNI space were converted to Talairach space using the Lancaster transform [41] as implemented in GingerALE. Coordinates from symptom provocation and cognitive-emotional tasks were first combined to examine the neural regions involved across tasks and then were analyzed separately to examine differences between the two design types. A replicate set of analyses was performed that included ROI-based studies. Differences in the whole-brain voxel-wise results with the inclusion of ROIs, when present, are noted in the tables and results.

For each analysis reported, peak activation coordinates were smoothed using a three-dimensional Gaussian filter and transformed into Gaussian probability distributions. These probability distributions were combined to generate whole-brain statistical maps of the ALE values on a voxel-wise basis. ALE statistics calculated the probability that at least one of the foci lay within each voxel and, therefore, the likelihood that each voxel was activated across all studies included in the analysis. The ALE statistic maps were compared with a null-distribution of random spatial associations between experiments (random-effects model) to assess for above chance clustering between experiments using a threshold at false discovery rate (FDR) corrected *P* < 0.05 and a cluster-extent of 100 mm^3^.

To explore the hypothesis that activity in the ventromedial prefrontal cortex and the amygdala was inversely related, we first identified whole-brain studies that reported increased ventromedial prefrontal cortex activity in controls relative to PTSD patients (which would suggest that this region was hypoactive in PTSD) and also reported regions of increased activity in PTSD relative to controls. Six studies were identified that met these criteria [1,11,12,21-23]. A meta-analysis was performed on the coordinates from these studies for the PTSD > Control contrast. Thus, we examined the regions that were hyperactive in PTSD when the ventromedial prefrontal cortex was hypoactive. Due to the small number of studies included, the analysis was thresholded at FDR corrected *P* < 0.05 and a less conservative cluster-extent of 40 mm^3^ (i.e., 5 contiguous voxels) was used.

## **Results**

Separate meta-analyses were run to examine the neural activity across and within symptom provocation and cognitive-emotional tasks in PTSD. Because of the variability in naming conventions of medial prefrontal cortex regions across different studies, activated regions are listed in the text and tables both by their structure specific name (e.g., medial frontal gyrus) and a general name signifying their contribution to a broader, less defined area (e.g., ventromedial prefrontal cortex which broadly includes the pregenual and subgenual anterior cingulate cortex, medial orbitofrontal cortex, and the ventral part of the medial prefrontal cortex).

### **Common activations for PTSD across tasks**

**Figure 1:**
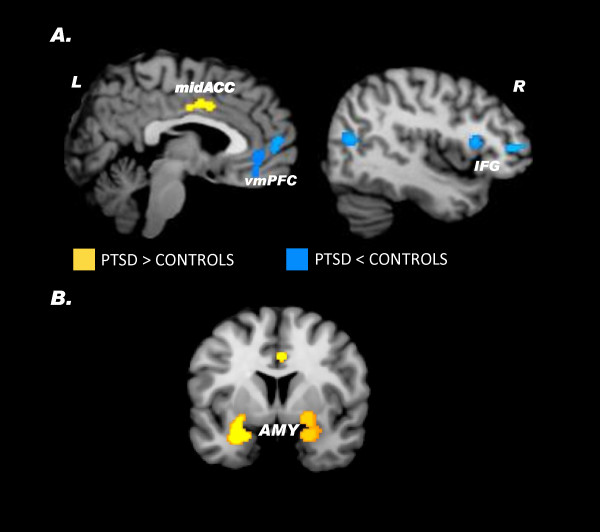
**A. Brain regions associated with PTSD across symptom provocation and cognitive-emotional tasks in the whole-brain voxel-wise analysis.B.** Bilateral amygdala activity is observed after including symptom provocation and cognitive-emotional ROI studies to the whole-brain voxel-wise results. Areas of hyperactivation in PTSD (PTSD > Control) are shown in yellow and areas of hypoactivation in PTSD (Control > PTSD) are shown in blue. Amy = amygdala, IFG = inferior frontal gyrus, L = left, midACC = mid anterior cingulate cortex, R = right, vmPFC = ventromedial prefrontal cortex.

The regions that were hyper- and hypoactive when studies were collapsed across task type (i.e., symptom provocation and cognitive-emotional) in PTSD relative to control subjects are reported in Table 3. We defined hyperactivity in PTSD as the results stemming from the PTSD > Control contrast and hypoactivity in PTSD as brain regions active from the Control > PTSD contrast. Patients with PTSD showed hyperactivation in the mid- and dorsal anterior cingulate (Figure 1A), left superior temporal gyrus, and left supplementary motor area. Robust bilateral amygdala and left dorsomedial prefrontal cortex activity was observed when ROI studies were included (Figure 1)B.

**Table 3:** Between-group comparison of activity across symptom provocation and cognitive-emotional studies

**Region**	**Hemisphere**	**Talairach**			**BA**	**Volume (mm**^**3**^**)**
		**x**	**y**	**z**		
**PTSD > Controls**						
**Whole Brain Analysis**						
Dorsal ACC	R	17.3	38.35	15.53	32	672
Mid/Dorsal ACC	R	3.49	−3.51	34.37	24	400
Superior Temporal Gyrus	L	−63.02	−47.37	18.13	22	152
Supplementary Motor Area	L	−22.02	−1.55	57.56	6	112
**Whole Brain + ROI Analysis**						
Amygdala	R	22.86	1.06	−13.32	--	1328
Amygdala	L	−25.95	−0.02	−17.87	--	1136
Dorsal ACC	R	17.33	38.35	15.44	32	640
Mid/Dorsal ACC	R	3.61	−3.18	34.29	24	288
Medial Frontal Gyrus (dmPFC)	R	1.08	31.44	37.87	8	216
Superior Temporal Gyrus	L	−63.15	−47.34	18.13	22	144
**Controls > PTSD**						
**Whole Brain Analysis**						
Medial Frontal Gyrus (vmPFC)	R	3.07	36.09	−6.69	11	984
Thalamus	L	−4.12	−14.12	17.89	--	480
Thalamus	R	12.06	−12.03	1.97	--	464
Inferior Frontal Gyrus	R	16.02	21.76	−12.36	47	440
Middle Occipital Gyrus	L	−31.42	−86.95	−1.35	18	392
Medial Frontal Gyrus	R	6.23	48.66	9.06	10	344
Middle Temporal Gyrus	R	45.03	−68.88	13.16	39	336
Inferior Frontal Gyrus	R	45.79	15.16	10.21	44	304
Precuneus	R	24.34	−56.37	38.59	7	280
Cerebellum	R	35.16	−82.16	−20	--	272
Medial Frontal Gyrus	R	11.89	42.19	24.43	9	272
Fusiform Gyrus	L	−51.87	−47.57	−15.78	37	256
Precuneus	R	25.76	−84.66	39.71	19	248
Superior Temporal Gyrus	L	−39.88	−23.07	5.88	13	144
Cerebellum	R	20	−47.03	−14.64	--	128
Inferior Frontal Gyrus	R	41.45	39.85	7.14	46	112
**Whole Brain** + **ROI Analysis**						
Pregenual ACC	R	5.17	44.99	14.35	32	1824
Medial Frontal Gyrus (vmPFC)	R	3.11	36.03	−6.34	11	864
Thalamus	L	−4.12	−14.11	17.88	--	480
Thalamus	R	12.01	−12.01	2	--	456
Inferior Frontal Gyrus	R	16.14	21.75	−12.28	47	424
Middle Occipital Gyrus	L	−31.35	−86.97	−1.34	18	384
Dorsal ACC	L	−6.43	9.14	25.01	24	368
Middle Temporal Gyrus	R	44.97	−68.82	13.1	39	328
Superior Frontal Gyrus	L	−23.94	50.88	8.8	10	256
Fusiform Gyrus	L	−51.75	−47.62	−15.77	37	248
Thalamus	L	−7.65	−6.83	7.93	--	248
Precuneus	R	24.44	−56.35	38.82	7	248
Inferior Frontal Gyrus	R	45.69	14.86	10.31	44	240
Precuneus	R	25.73	−84.68	39.86	19	216
Cerebellum	R	35.01	−82	−20	--	208
Superior Temporal Gyrus	L	−39.98	−22.77	5.98	13	120

*P* < .05 FDR-corrected, cluster activation extent ≥ 100 mm^3^. *ACC* = anterior cingulate cortex, *BA* = Brodmann area, *dmPFC* = dorsomedial prefrontal cortex, *L* = left, *R* = right, *vmPFC* = ventromedial prefrontal cortex.

Notably, there were several regions of hypoactivation in PTSD relative to controls including the medial frontal gyrus (ventromedial prefrontal cortex; Figure 1B), thalamus, right inferior frontal gyrus (Figure 1B), and right middle temporal gyrus. When ROI studies were included, the results remained consistent with additional activity observed in the pregenual anterior cingulate cortex (Table 3).

### **Symptom provocation studies**

**Figure 2:**
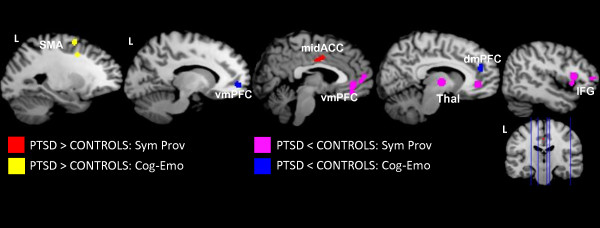
**Overlay of brain regions associated with PTSD by task design.** Warm colors indicate regions of hyperactivity in PTSD patients during symptom provocation study designs (red) and cognitive-emotional study designs (yellow). Cool colors indicate regions of hypoactivity in PTSD during symptom provocation designs (purple) and cognitive-emotional designs (blue). Cog-Emo = Cognitive-emotional, DmPFC = dorsomedial prefrontal cortex, IFG = inferior frontal gyrus, L = left, midACC = mid anterior cingulate cortex, SMA = supplementary motor area, Sym Prov = symptom provocation, Thal = thalamus, vmPFC = ventromedial prefrontal cortex.

A meta-analysis of symptom provocation designs was conducted to reveal the regions that were involved in reliving one’s traumatic event (Table 4). The regions consistently hyperactivated in PTSD were the mid- and dorsal anterior cingulate cortex. By contrast, widespread hypoactivity was observed, including the medial frontal gyrus (ventromedial prefrontal cortex), right inferior frontal gyrus, and right precuneus. These results were unchanged with the inclusion of ROI studies. Figure 2 displays brain activation separately for symptom provocation and cognitive-emotional studies.

**Table 4:** Between-group comparison results of symptom provocation articles and cognitive-emotional articles

**Region**	**Hemisphere**	**Talairach**			**BA**	**Volume (mm**^**3**^**)**
		**x**	**y**	**z**		
**SYMPTOM PROVOCATION**						
**PTSD > Controls**						
**Whole Brain Analysis**						
Dorsal ACC	R	15.38	37.69	16.96	32	344
Mid/Dorsal ACC	R	2.65	−8.91	33.77	24	296
Mid/Dorsal ACC	R	0.3	−19.11	37.4	24	104
**Whole Brain** + **ROI Analysis**						
Dorsal ACC	R	15.38	37.69	16.96	32	344
Mid/Dorsal ACC	R	2.65	−8.91	33.77	24	296
**Controls > PTSD**						
**Whole Brain Analysis**						
Medial Frontal Gyrus (vmPFC)	R	3.32	36.07	−6.42	11	1152
Thalamus	R	12	−12	2	--	648
Thalamus	L	−4.02	−14.02	17.98	--	648
Inferior Frontal Gyrus	R	45.62	14.54	8.65	44	480
Precuneus	R	25.83	−84.66	39.57	19	296
Inferior Frontal Gyrus	R	41.77	40.46	7.18	46	216
Medial Frontal Gyrus	R	3.94	50.11	7.37	10	208
**Whole Brain** + **ROI Analysis**						
Medial Frontal Gyrus (vmPFC)	R	3.34	36.13	−6.34	11	1168
Thalamus	R	12	−12	2	--	648
Thalamus	L	−4.03	−14.01	17.97	--	648
Medial Frontal Gyrus	R	0.63	47.89	5.66	10	504
Inferior Frontal Gyrus	R	45.69	14.62	8.52	44	464
Precuneus	R	25.83	−84.66	39.57	19	296
Inferior Frontal Gyrus	R	41.77	40.46	7.18	46	216
**COGNITIVE- EMOTIONAL**						
**PTSD > Controls**						
**Whole Brain Analysis**						
Supplementary Motor Area	L	−22.13	−1.49	57.27	6	128
Supplementary Motor Area	L	−25.39	2.02	40.31	6	104
**Whole Brain** + **ROI Analysis**						
Amygdala	R	22.93	1.03	−13.28	--	1376
Amygdala	L	−27.21	−0.16	−17.37	--	840
Medial Frontal Gyrus (dmPFC)	R	0.98	31.46	37.87	8	224
**Controls > PTSD**						
**Whole Brain Analysis**						
Pregenual ACC (vmPFC)	L	−13.93	49.87	−2.12	32	400
Medial Frontal Gyrus (dmPFC)	R	10.63	40.6	23.03	9	272
**Whole Brain + ROI Analysis**						
Medial Frontal Gyrus (dmPFC)	R	11.17	41.96	21.25	9	984
Pregenual ACC (vmPFC)	L	−13.97	49.76	−2.16	32	384

*P* < 0.05 FDR-corrected, activation extent ≥ 100 mm^3^. *ACC* = anterior cingulate cortex, *BA* = Brodman area, *dmPFC* = dorsomedial prefrontal cortex, *L* = left, *R* = right, *vmPFC* = ventromedial prefrontal cortex.

### **Cognitive-emotional studies**

Cognitive-emotional studies included stimuli that were negative, but not trauma-specific (e.g., fearful faces). The whole-brain voxel-wise analysis revealed hyperactivity in supplementary motor area. Bilateral amygdala and medial frontal gyrus (dorsomedial prefrontal cortex, BA 8) activity was observed when ROI studies were included (see Table 4). Regions of hypoactivity (Figure 2) included the pregenual anterior cingulate cortex (ventromedial prefrontal cortex) and medial frontal gyrus (dorsomedial prefrontal cortex, BA 9).

### **Ventromedial prefrontal cortex meta-analysis**

We next performed a meta-analysis on regions that were hyperactive in PTSD within studies that reported decreased ventromedial prefrontal cortex activity (see Methods). The analysis showed that when the ventromedial prefrontal cortex was hypoactivated, greater amygdala activation was observed in PTSD, supporting the hypothesis that activity in the ventromedial prefrontal cortex and amygdala are inversely related. Other regions that showed increased activity included the right middle and inferior temporal gyrus, left superior temporal gyrus, bilateral precuneus, and right putamen (Table 5).

**Table 5:** Ventromedial prefrontal cortex meta-analysis results

**Region**	**Hemisphere**	**Talairach**			**BA**	**Volume (mm**^**3**^**)**
		**x**	**y**	**z**		
**PTSD > Controls**						
Middle Temporal Gyrus	R	49.54	−36.22	−12.82	20	136
Amygdala	L	−19.01	−0.97	−19.03	--	64
Precuneus	L	−12.98	−53.03	33.02	31	64
Putamen	R	19.43	1.15	−7.72	--	56
Cerebellum	R	36.64	−65.35	−37.02	--	48
Amygdala	R	19.35	0.65	−18.99	--	48
Superior Temporal Gyrus	L	−62.66	−48.01	18.66	22	48
Precuneus	R	20.65	−76.99	40.66	7	48
Supplementary Motor Area	L	−47.98	4.01	41.66	6	48
Inferior Temporal Gyrus	R	54.78	−45.22	−11.61	20	40

*P* < .05 FDR-corrected, activation extent ≥ 40 mm^3^. *BA* = Brodmann area, *L* = left, *R* = right.

## Discussion

The present study used quantitative meta-analysis to examine the pathophysiology of PTSD. The results confirmed involvement of a subset of regions implicated in fear-circuitry models of PTSD, including robust hyperactivity in the dorsal anterior cingulate cortex, hypoactivity in the ventromedial prefrontal cortex in PTSD, and an inverse relationship between activity in the ventromedial prefrontal cortex and amygdala. However, additional regions were found to be hyper- and hypoactive in PTSD, suggesting that a broader view of the neural circuitry of PTSD should be considered. Collapsing across symptom provocation and cognitive-emotional studies, the whole-brain voxel-wise analysis revealed hyperactivation of the mid/dorsal anterior cingulate cortex, supplementary motor area, and superior temporal gyrus in PTSD. These regions have been previously shown to be part of a putative ‘salience network’ that processes autonomic, interoceptive, homeostatic, and cognitive information of personal relevance [42,43]. Ultimately, the salience network helps an organism evaluate whether stimuli in the environment should be approached or avoided. Importantly, activity in this salience network is positively correlated with anxiety [43]. We propose that in PTSD, the behavioral manifestation of increased output of the salience network may provide privileged cognitive resources to a broad range of salient stimuli leading to hypervigilance and disruption of goal-directed activity. This notion is consistent with observations in PTSD patients of deficits in working memory for not only trauma-related negative distractors, but also neutral distractors [44], suggesting that a variety of stimuli become potentially salient for patients with PTSD. From this viewpoint, negative emotions other than fear can be associated with the disorder, as long as they are salient and associated with a stress response.

The dorsal anterior cingulate cortex is a key node in the salience network. Earlier conceptualizations of the region suggested that its role was primarily in “cold” cognitive processes, in contrast to the ventral aspects of the anterior cingulate cortex that were thought to be involved in affective processing [45]. However, more recent data have not corroborated a cognitive versus affective dissociation. Recent reviews have called attention to the involvement of the dorsal anterior cingulate cortex in PTSD [46,47], which may subserve learned fear, fear appraisal and expression, and sympathetic activity [48]. More broadly, dorsomedial prefrontal regions (including the dorsal anterior cingulate cortex) have been associated with appraisal and evaluation whereas ventromedial prefrontal regions are associated with regulatory functions. This dissociation is consistent with the findings reported here, where more dorsal prefrontal regions, including the dorsomedial prefrontal cortex and mid/dorsal anterior cingulate cortex were active in patients with PTSD and may suggest heightened appraisals of potential threats in the environment, whereas hypoactivity in ventromedial prefrontal regions may reflect dysfunction in emotion regulation.

Interestingly, the present results highlight the contribution of the mid-cingulate in PTSD, adding to the growing evidence that this region plays an important role in this disorder [49-51] and may be important for fear conditioning [52]. The dorsal anterior cingulate spans a large area, encompassing BAs 24, 32, and 33. Whereas a more anterior portion of the dorsal anterior cingulate was activated in both PTSD patients and control subjects in the present meta-analysis, a more posterior region was hyperactivated only in PTSD. A previous study demonstrated that individuals with severe PTSD symptomatology activated the mid/dorsal anterior cingulate to a greater extent than controls during an emotional oddball task, suggesting that distracting stimuli are given attentional preference at the expense of a goal-relevant task in PTSD [49]. These findings provide converging evidence for the role of the mid/dorsal anterior cingulate cortex in salience processing. Another region in the salience network, the amygdala, was observed only when using a less stringent spatial extent in the whole-brain analysis or when considering ROI analyses. The amygdala is notoriously difficult to image due to vulnerability to susceptibility artifact and its relatively small volume, which could account for lack of robust findings in the whole-brain analysis. Alternatively, it is possible that the amygdala is not as central of a region in PTSD as current neurocircuitry models suggest, consistent with previous meta-analysis data showing that the amygdala is more frequently active in patients with social anxiety disorder and specific phobia than PTSD [37].

With the addition of ROI analyses, amygdala activity was observed for cognitive-emotional tasks but not symptom provocation tasks, suggesting that the type of task employed within a study influences amygdala activity in PTSD. There is emerging recognition that the amygdala may play a more general role in processing ambiguous and salient stimuli in the environment [53-55], of which fear may be one particularly potent instance. The amygdala, which is composed of several distinct but highly interconnected nuclei, is not specific to fear states but is also activated for unusual and novel stimuli [56] and unpredictability [57]. Therefore, the stimuli and study designs employed during cognitive-emotional studies of PTSD, which often present novel and ambiguous stimuli intermittently, may evoke more central involvement of the amygdala than autobiographical trauma scripts, which were often familiar and unambiguous from the start. Other explanations for the lack of amygdala activity in symptom provocation designs are less likely. Both the symptom provocation designs and the cognitive-emotional ROI studies (in which amygdala activity was observed most robustly) were block designs; therefore, the results are unlikely to be attributable to differences in neuroimaging experimental design (i.e., event-related vs. block designs). Furthermore, the majority of both symptom provocation and cognitive-emotional studies were fMRI rather than PET, suggesting that the difference is not due to imaging modality. The discrepancy in amygdala activity for cognitive-emotional and symptom provocation studies underscores the importance of considering the cognitive task when interpreting activation differences (or lack thereof) in the amygdala in PTSD and control participants.

In the present study, widespread hypoactivity in prefrontal cortex in PTSD was observed, including both medial and lateral regions. Notably, hypoactivity in the ventromedial prefrontal cortex was present in both symptom provocation and cognitive-emotional study designs. To examine the relationship between the ventromedial prefrontal cortex and amygdala, we performed a meta-analysis that identified regions of hyperactivity within a subset of studies that showed a decrease in ventromedial prefrontal cortex activity in PTSD patients. We reasoned that under conditions of diminished ventromedial prefrontal cortex activity, which may signify reduced top-down governance of interconnected regions, we would observe greater amygdala activity. The results showed that when the ventromedial prefrontal cortex was hypoactive, the amygdala, putamen, and temporal cortex were hyperactivated. These results support the notion that a consequence of hypoactivity of the ventromedial prefrontal cortex may be greater responsivity of the amygdala in the face of negative information. Although the direction of this effect cannot be determined conclusively because the neural connections between the amygdala and ventromedial prefrontal cortex are bidirectional, there is a well-established literature showing the involvement of ventromedial prefrontal cortex in regulatory control across species [58]. It is important to note that the ventromedial prefrontal cortex is not a single entity, but rather is composed of multiple distinct regions (i.e., subgenual and pregenual anterior cingulate cortex, medial portions of orbitofrontal gyrus, and medial frontal gyrus) that subserve a variety of functions. For instance, the non-human animal literature suggests that bordering divisions within ventromedial prefrontal cortex may be responsible for both inhibition and facilitation of autonomic arousal [58]. This may help to explain why some studies of PTSD show increased activation in this region [59] and suggests that a more fine-grained analysis is required to better elucidate the various functions of the ventromedial prefrontal cortex. Nevertheless, the results of the current meta-analysis show robust hypoactivation in the ventromedial prefrontal cortex consistent across task type, underscoring its hypothesized role in regulatory control.

Importantly, additional prefrontal cortex regions such as the inferior frontal gyrus were hypoactivated in PTSD. This finding is notable as previous work has implicated the role of inferior frontal gyrus in emotion regulation, including inhibition from emotional distraction [60] and emotional thought suppression [61]. Moreover, the inferior frontal gyrus is purported to be involved in a network of lateral prefrontal cortex regions involved in changing one’s negative thoughts to reduce the impact of negative feelings (i.e., cognitive reappraisal) [62]. Although speculative, it is possible that decreased activity in lateral prefrontal cortex may reflect PTSD patients’ difficulty challenging negative thoughts to cope with emotional stimuli. Contemporary psychological models of PTSD highlight the role of negative appraisals and emotion regulation in the etiology and maintenance of PTSD. One of the most successful psychosocial interventions for PTSD, cognitive processing therapy, is based upon the notion that faulty cognitions and interpretation surrounding the traumatic event interferes with the natural recovery process after a trauma [63]. For example, a female rape victim who misattributes blame to herself for attending a party where the rape occurred may then mistrust her decisions in every aspect of her life, leading to experiential avoidance and withdrawal from social relationships. Research has supported the notion that negative self-appraisals are associated with PTSD symptom maintenance [64] and therefore the DSM-V may now include the presence of negative cognitions as a core feature of the disorder [35]. Cognitive processing therapy encourages the patient to adopt a more balanced view of the circumstances surrounding the traumatic event, as well as current personal events by challenging negative thoughts. Given the present results, future studies should examine whether individuals who benefitted from cognitive processing therapy recruit the inferior frontal gyrus to a greater extent compared to pre-therapy, as well as compared to individuals who did not benefit from therapy.

### **Limitations**

A constraint of the current study is the availability of studies that met our criteria for inclusion into the analyses. Although the literature search started with 79 studies, the exclusion of studies that did not include stereotaxic coordinates likely reduced our power to detect less robust activations. Although the number of foci included in this study is more than the minimum recommended for a meta-analysis, it remains an open question whether a larger sample will reveal additional networks central to the PTSD diagnosis. For example, amygdala activity was observed in the PTSD group only when considering ROI analyses or using a less stringent spatial extent. Therefore, the limited number of studies available for the meta-analysis may have had an impact on the ability to detect amygdala activity within the whole-brain analysis. Activity in another key node within the salience network, the anterior insula, was observed in the PTSD group using a less stringent cluster threshold (FDR corrected, *P* < 0.05, cluster-extent = 24 mm^3^). Future studies could isolate resting-state networks as a more powerful and robust method towards understanding the functional connections between nodes of the salience network in PTSD. Interestingly, a recent resting-state study in PTSD revealed greater connectivity between the amygdala and insula in patients with PTSD than trauma-exposed controls [65]. The results are consistent with the notion that key nodes within the salience network are highly coactive in PTSD and may underlie the hallmark symptoms of the disorder.

Although there is convincing evidence that the hippocampus becomes dysfunctional as a result of chronic stress [66] and activity in this region has shown to be negatively correlated with arousal symptoms in PTSD [67], hippocampal activity was not observed in the present meta-analysis making it unclear how this region contributes to neurocircuitry models of PTSD. Many of the tasks included in this meta-analysis were not optimal for eliciting hippocampal activity and those that do examine hippocampal function in PTSD show mixed results. There is a growing functional neuroimaging literature examining learning and memory in PTSD, which may clarify the role of hippocampus given that these types of paradigms traditionally activate the hippocampus in healthy individuals.

Finally, working with a limited sample required inclusion of studies with patients on medication and/or co-morbid depression. As additional studies are published and software development continues, future meta-analyses may be able to focus exclusively on PTSD or include depression and medication status as covariates in the analyses.

## Conclusions

The goal of the present meta-analysis was to examine the neurocircuitry of PTSD by considering a set of studies that were diverse in terms of functional imaging modality, study design, and PTSD trauma type. The results provide evidence for hyperactivation of regions important for vigilance and salience detection, and hypoactivation of regulatory networks engaged in regulation of autonomic arousal and cognition. The key salience network regions that appear to be important in PTSD include the dorsomedial prefrontal cortex (including mid/dorsal anterior cingulate cortex), supplementary motor area, and superior temporal gyrus.

Furthermore, regulatory control regions include two primary networks that appear to be dysfunctional in PTSD, including ventromedial prefrontal cortex control over the amygdala and lateral prefrontal regions putatively involved in modification of thought and inhibition of distracting emotions. This model is consistent with the findings that therapies designed to both extinguish fear responses and promote emotion regulation through challenging negative cognitions are helpful for the treatment of PTSD.

## Competing interests

The author(s) declare that they have no competing interests.

## Authors’ contributions

Ms. Amanda Mikedis conducted a literature review, performed data analysis, and assisted in writing the Methods section. Dr. Jasmeet Hayes and Dr. Scott Hayes helped with the literature review and data analysis, and wrote the paper. All authors read and approved the final manuscript.
